# Racial/Ethnic Disparities in Anticoagulation for Atrial Fibrillation by Sex and Within High and Low Stroke Risk Populations

**DOI:** 10.19102/icrm.2025.16062

**Published:** 2025-06-15

**Authors:** William J. Tate, Darius White, Grace Ha, James Alzate, Dolphurs Hayes, Leon M. Ptaszek, Jeremy Ruskin, Joseph R. Betancourt, Oyere Onuma, Jason H. Wasfy, Malissa J. Wood, Moussa Mansour

**Affiliations:** 1 Department of Medicine, Massachusetts General Hospital, Boston, MA, USA; 2 Department of Medicine, Division of Cardiology, Massachusetts General Hospital, Boston, MA, USA

**Keywords:** Anticoagulation, atrial fibrillation, healthcare disparity, racial disparity, sex disparity

## Abstract

Atrial fibrillation (AF) increases the risk of thromboembolic stroke, and oral anticoagulants (OACs) are an effective tool to reduce this risk. Previous studies have demonstrated that female, black, Hispanic, and Asian groups are less likely to be prescribed OACs. This study explores OAC rates by racial/ethnic group and assesses differences within sexes and between high and low CHA_2_DS_2_-VASc risk groups. Using a database of AF patients, we employed logistic regression models to assess the association between race/ethnicity and OAC rates among all individuals and according to CHA_2_DS_2_-VASc risk and sex subgroups. Black, Hispanic, and Asian individuals with AF had lower OAC rates compared to white individuals (adjusted odds ratio [aOR], 0.84; 95% confidence interval [CI], 0.77–0.91) (aOR, 0.92; 95% CI, 0.85–0.99) (aOR, 0.80; 95% CI, 0.72–0.88). Female patients with AF had lower OAC rates than male patients (aOR, 0.66; 95% CI, 0.64–0.68). Among male patients, black, Hispanic, and Asian patients had lower OAC rates while, among female patients, only black patients had a lower OAC rate. In the low-risk CHA_2_DS_2_-VASc group, only Asian individuals had a lower OAC rate compared to white individuals, while, in the high-risk group, this trend was observed only for black individuals. Women, particularly black women, are less likely to receive OACs compared to men and their white counterparts. High-risk black individuals face reduced OAC use, while low-risk white individuals have high OAC rates. Subjective decision-making may contribute to these disparities, with the most significant disparities observed in black individuals, particularly black women. This “double hit” affecting black women could be the target of equity-focused interventions.

## Introduction

Atrial fibrillation (AF) is the most common arrhythmia worldwide and is associated with an increased risk of thromboembolic stroke and overall mortality.^1–3^ Despite having a reported lower incidence of AF, black individuals have worse outcomes related to AF compared to white individuals.^4,5^ Similarly, female patients with AF experience greater overall mortality, increased cardiovascular mortality, a higher risk of stroke, and more cardiac events compared to male patients with AF.^6^ One of the mainstays of treatment for AF is thromboembolic stroke risk reduction with oral anticoagulants (OACs).^7^ Several previous studies have demonstrated that there are both racial and sex disparities in the use of OACs for AF.^8–10^ Similarly, studies have shown that both racial and sex disparities have persisted despite the increasing availability of direct OACs (DOACs).^8,11^ However, there are some studies that have not found a sex disparity in OAC use among high-risk CHA_2_DS_2_-VASc patients.^12^ In general, few studies have explored how CHA_2_DS_2_-VASc stroke risk influences OAC use by race/ethnicity, and there do not appear to be any studies looking specifically at race/ethnicity OAC use within sex categories. Of studies that do explore race/ethnicity in OAC use in AF, most are only able to compare two or three racial/ethnic groups. In this study, we sought to explore if racial/ethnic differences are seen in OAC use and specifically whether CHA_2_DS_2_-VASc stroke risk influences OAC rates between white, black, Hispanic, and Asian groups. Second, we aimed to determine if OAC rates differ by race/ethnicity within sex categories. Finally, we aimed to explore whether the use of DOACs differs between racial/ethnic groups.

## Methods

The analytic methods, statistical code, and data that support the findings of this study will be made available to other researchers upon reasonable request for the purposes of reproducing the results or replicating the procedure. These requests can be made via email to the corresponding author.

For this study, a novel registry database of patients with a diagnosis of AF was created using common Epic rules (CER) across the electronic medical record of the Mass General Brigham Hospital system in both the inpatient and outpatient settings. Mass General Brigham–affiliated locations in this study included Massachusetts General Hospital, Brigham and Women’s Hospital, Brigham and Women’s Faulkner Hospital, McLean Hospital, Newtown-Wellesley Hospital, Spaulding Rehabilitation Hospital, Salem Hospital, Wentworth-Douglass Hospital, Martha’s Vineyard Hospital, Cooley Dickinson Hospital, and Nantucket Cottage Hospital. Patients with AF were identified using the following criteria: an active International Classification of Diseases, Tenth Revision, code of AF in the problem list, a documented medical history of AF, or an encounter diagnosis of AF within the past 365 days. For patients who met registry inclusion criteria, the most recent status of several pre-specified demographic and clinical characteristics was extracted. This study was approved by the local institutional review board.

This was a cross-sectional study reflecting data extracted in October 2022. The study group selected from the database was restricted to individuals between the ages of 18 and 110 years. All registry individuals with missing data for any of the study outcomes or covariates, except for body mass index (BMI), were excluded from the study population.

The primary study outcome was the rate of OAC prescription. An OAC prescription was defined as an active order in the medication list for any of the following medications: warfarin, apixaban, rivaroxaban, dabigatran, or edoxaban. A second outcome measure was the rate of DOAC prescription. A DOAC prescription was defined as an active order in the medication list for any of the following medications: apixaban, rivaroxaban, dabigatran, or edoxaban.

The primary independent variable in this study was race/ethnicity. Database variables extracted from Epic included self-reported race and ethnicity as separate entities. A variable race/ethnicity consisting of mutually exclusive categories of white, black, Hispanic, and Asian was created following established Executive Office of Budget and Management standards for the classification of federal data on race and ethnicity.^13^ Following these rules, if a patient was classified under the ethnicity “Hispanic or Latino,” then their race/ethnicity was categorized as Hispanic regardless of their racial group. As such, a patient with black race and Hispanic ethnicity was categorized under the race/ethnicity group Hispanic. This allowed authors to compare Hispanic as an individualized racial/ethnic group, eliminating the need to denote other racial groups as non-Hispanic.

**Supplementary Figure S1:**
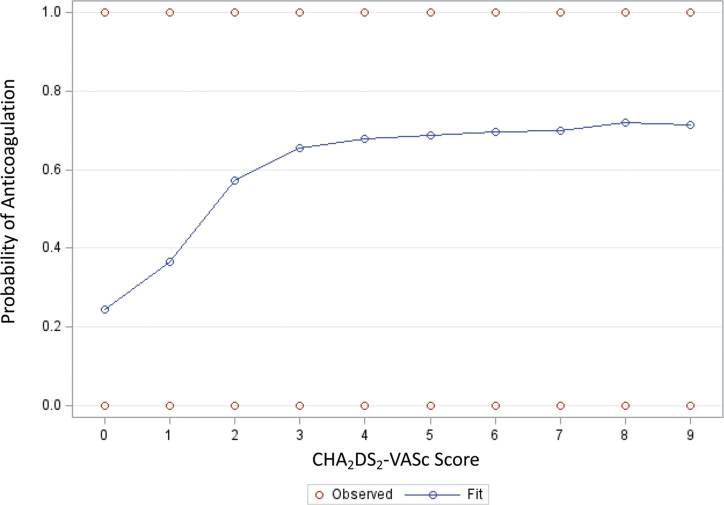
Effect plot of receiver operating characteristic curve showing predicted probabilities of being on anticoagulation for a value of CHA_2_DS_2_-VASc.

Other important independent variables included age, sex, BMI, insurance type, CHA_2_DS_2_-VASc score, and HAS-BLED score. Sex was classified as male or female as categorized by Epic, with male as the referent group. Given the constraints of CER, the spectrum of gender identity is not captured in this dataset. As such, male/men and female/women are used interchangeably to signify biological sex. BMI from the most recent encounter within the preceding 3 years was used. In cases where there was no BMI recorded within the preceding 3 years, this variable was listed as missing. Insurance status was classified as private, Medicare, or Medicaid, with private as the referent group. Individuals dual-enrolled in Medicare with a private supplement plan were classified as Medicare recipients. A CHA_2_DS_2_-VASc stroke risk score was calculated with 1 point for each of the following, unless otherwise noted: congestive heart failure, hypertension, age ≥ 65 years, age ≥ 75 years, diabetes, stroke or transient ischemic attack or thromboembolism (2 points), vascular disease, and female sex.^14^ An effect plot from a receiver operating characteristic curve was created to determine the probability of being prescribed an OAC by CHA_2_DS_2_-VASc **(Supplementary Figure S1)**. Based on this receiver operating characteristic curve effect plot, individuals were stratified into CHA_2_DS_2_-VASc low (0–1) or high (2–9) stroke risk groups, which aligned with society guidelines for anticoagulation in AF.^7^ A HAS-BLED score was calculated with 1 point for each of the following: hypertension, abnormal liver function, abnormal renal function, stroke history, bleeding history or predisposition, labile international normalized ratio, age ≥ 65 years, medication usage predisposing to bleeding, and alcohol use of greater than or equal to eight standard drinks per week.^15^

Baseline patient demographics were characterized by race/ethnicity using percent for categorical variables and median with interquartile range (IQR) values for continuous variables. Categorical variables were analyzed using the chi-squared test, and continuous variables were analyzed using the Wilcoxon non-parametric test.

Logistic regression models were created to assess the association between race/ethnicity and OAC. Covariates used in risk adjustment included age, sex, insurance type, CHA_2_DS_2_-VASc, and HAS-BLED. Additional models were created to assess the association between race/ethnicity and OAC when stratified by sex and low- and high-risk CHA_2_DS_2_-VASc groups. A logistic regression model was also created to assess the association between race/ethnicity and the use of DOAC, with covariates used in risk adjustment, including age, sex, insurance type, and BMI. In cases of missing BMI data, a single imputation method was used with the average BMI for each race/ethnicity–sex category. All other cases with missing data were excluded from analysis. A sensitivity analysis was performed without insurance status to ensure that multicollinearity was not influencing results **(Supplementary Table S1)**. All tests were two-tailed, with statistical significance set at *P* < .05. All analyses were performed using SAS version 9.4 (SAS institute, Cary, NC, USA).

**Supplementary Table S1:** Sensitivity Analysis of Association Between Race/Ethnicity and Use of Oral Anticoagulation Without Adjustment for Insurance Status

Race/Ethnicity	No. of Patients Using OACs (%)	OR	95% CI	*P* Value
White (n = 101,262)	63,251 (62.5%)	1.00	(Reference)	—
Black (n = 2737)	1535 (56.1%)	0.81	0.75–0.88	<.001
Hispanic (n = 3168)	1795 (56.7%)	0.89	0.82–0.95	.001
Asian (n = 1832)	1036 (56.6%)	0.78	0.71–0.86	<.001

*Abbreviations:* CI, confidence interval; OAC, oral anticoagulant; OR, odds ratio. Logistic regression adjusted for age, sex, CHA_2_DS_2_-VASc, and HAS-BLED.

## Results

At the time of analysis in October 2022, the registry database contained 118,727 individuals. After exclusion, a total of 108,999 individuals with AF were included in the analysis, of whom 101,262 (92.9%) identified as white, 2737 (2.5%) identified as black, 3168 (2.9%) identified as Hispanic, and 1832 (1.7%) identified as Asian. The population was predominantly male (58.6%) and had a median age of 75 years (IQR, 67–83 years). Baseline demographic and clinical characteristics of the study population are shown in **Table 1**. Differences between race/ethnicity were seen according to age, sex, insurance type, BMI, CHA_2_DS_2_-VASc, HAS-BLED, OAC prescription rate, and DOAC prescription rate. White individuals were older and more likely to have Medicare insurance than black, Hispanic, and Asian groups. There were significant differences between race/ethnicity for age within low- and high-risk CHA_2_DS_2_-VASc groups **(Supplementary Table S2)**. A total of 9728 individuals were excluded from the analysis. Eight individuals were excluded due to missing sex data, 1570 individuals were excluded for missing age data, 164 individuals were excluded after age restrictions were applied, and 5484 individuals were excluded for missing race/ethnicity data. There were 2502 individuals with missing insurance data, of whom 2291 identified as white, 86 identified as black, 79 identified as Hispanic, and 46 identified as Asian. There were 14,279 individuals with missing BMI data, which were included in the analysis as outlined in the methods.

**Table 1:** Baseline Characteristics by Race/Ethnicity

	White, n = 101,262 (92.9%)	Black, n = 2737 (2.5%)	Hispanic, n = 3168 (2.9%)	Asian, n = 1832 (1.7%)	*P* Value
Demographic characteristics					
Female sex, n (%)	41,664 (41.1%)	1242 (45.4%)	1473 (46.5%)	779 (42.5%)	<.001
Age, median (IQR), years	76 (67–83)	69 (60–79)	70 (58–79)	74 (64–82)	<.001^a^
Age, n (%), years					
<65	18,691 (18.5%)	998 (36.5%)	1188 (37.5%)	483 (26.4%)	
65–74	27,579 (27.2%)	779 (28.5%)	809 (25.5%)	468 (25.6%)	—
≥75	54,992 (54.3%)	960 (35.0%)	1171 (37.0%)	881 (48%)	
Insurance, n (%)					
Private	24,703 (24.4%)	940 (34.4%)	1068 (33.7%)	634 (34.6%)	
Medicare	74,930 (74.0%)	1528 (55.8%)	1566 (49.4%)	1068 (58.3%)	<.001
Medicaid	1629 (1.6%)	269 (9.8%)	534 (16.9%)	130 (7.1%)	
BMI, median (IQR)	27.8 (24.3–32.2)	29.0 (24.7–34.3)	29.3 (25.6–33.8)	24.6 (22.2–27.4)	<.001^a^
Clinical characteristics					
CHA₂DS₂-VASc score, median (IQR)	4 (2–5)	4 (2–5)	3 (2–5)	4 (2–5)	.007^a^
CHA₂DS₂-VASc risk group, n (%)					
Low risk	14,300 (14.1%)	451 (16.5%)	653 (20.6%)	338 (18.5%)	—
High risk	86,962 (85.9%)	2286 (83.5%)	2515 (79.4%)	1494 (81.5%)	
HAS-BLED score, median (IQR), points	2 (2–3)	2 (1–3)	2 (1–3)	2 (1–3)	<.001^a^
Anticoagulation					
Patient on anticoagulation, n (%)	63,251 (62.5%)	1535 (56.1%)	1795 (56.7%)	1036 (56.6%)	<.001
DOAC, n (%)	50,339 (79.6%)	1203 (78.4%)	1462 (81.4%)	864 (83.4%)	.002
Warfarin, n (%)	12,912 (20.4%)	332 (21.6%)	333 (18.6%)	172 (16.6%)	.002

*Abbreviations:* BMI, body mass index; DOAC, direct oral anticoagulant; IQR, interquartile range. ^a^Wilcoxon non-parametric test.

**Supplementary Table S2:** CHA₂DS₂-VASc Group by Age and Race/Ethnicity

	White	Black	Hispanic	Asian	*P* Value
Low-risk CHA₂DS₂-VASc, age (years), median (IQR)	60 (52–64)	51 (39–60)	50 (38–58)	54 (45–62)	<.001
High- CHA₂DS₂-VASc, age (years), median (IQR)	78 (71–85)	72 (64–80)	73 (65–81)	77 (70–84)	<.001

*Abbreviation:* IQR, interquartile range.

**Figure 1:**
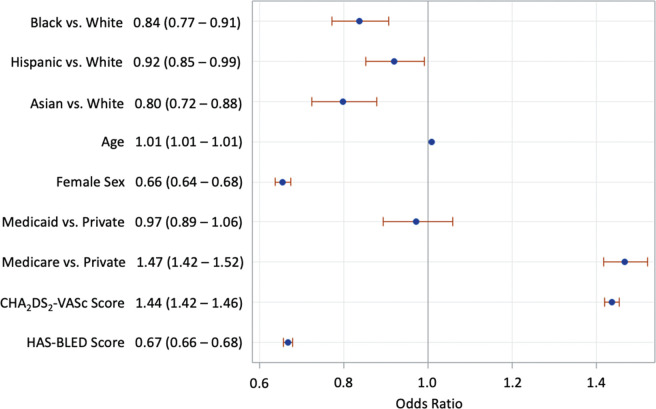
Odds ratio plot with 95% confidence intervals for adjusted logistic regression of being on anticoagulation by race/ethnicity adjusted for age, sex, insurance type, CHA_2_DS_2_-VASc score, and HAS-BLED score.

Black (adjusted odds ratio [aOR], 0.84; confidence interval [CI], 0.77–0.91), Hispanic (aOR, 0.92; 95% CI, 0.85–0.99), and Asian (aOR, 0.80; 95% CI, 0.72–0.88) individuals with AF were less likely to be prescribed OACs compared to white individuals **(Table 2)**. Female patients (aOR, 0.66; 95% CI, 0.64–0.68) were less likely to be prescribed OACs when compared to male patients. Individuals with Medicare insurance were more likely to be prescribed an OAC compared to those with private insurance, while those with Medicaid showed no difference compared to those with private insurance even after adjustment for age. Both age and CHA_2_DS_2_-VASc score were associated with increased rates of OAC prescription, while HAS-BLED score was associated with decreased rates of OAC prescription **(Figure 1)**. In a regression analysis, insurance status had a variance inflation factor of 1.282. To ensure that co-linearity was not biasing results, a sensitivity analysis was performed without insurance status, resulting in similar outcomes to that seen in the original full logistic regression analysis **(Supplementary Table S1)**.

**Table 2:** Association Between Race/Ethnicity and Use of Oral Anticoagulation

Race/Ethnicity	No. of Patients Using OACs (%)	Unadjusted Logistic Regression			Adjusted Logistic Regression		
		OR	95% CI	*P* Value	OR	95% CI	*P* Value
White (n = 101,262)	63,251 (62.5%)	1.00	(Reference)	—	1.00	(Reference)	—
Black (n = 2737)	1535 (56.1%)	0.77	0.71–0.83	<.001	0.84	0.77–0.91	<.001
Hispanic (n = 3168)	1795 (56.7%)	0.80	0.74–0.85	<.001	0.92	0.85–0.99	.0321
Asian (n = 1832)	1036 (56.6%)	0.79	0.72–0.87	<.001	0.80	0.72–0.88	<.001

*Abbreviations:* CI, confidence interval; OAC, oral anticoagulant; OR, odds ratio. Logistic regression adjusted for age, sex, insurance type, CHA_2_DS_2_-VASc, and HAS-BLED.

**Figure 2:**
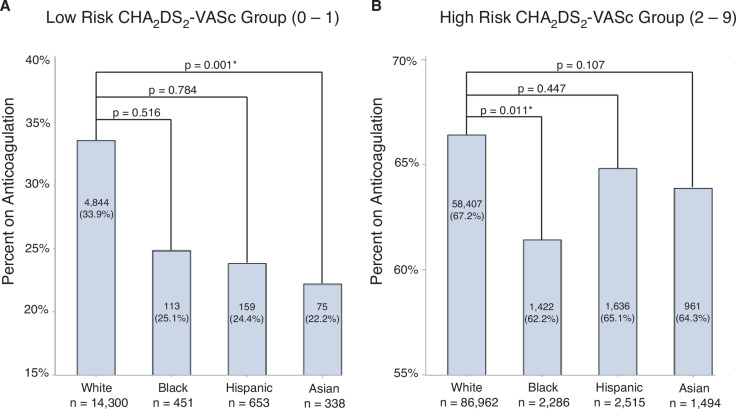
**A:** Bar graph comparing percent on anticoagulation by race/ethnicity in the low-risk CHA_2_DS_2_-VASc group. **B:** Bar graph comparing percent on anticoagulation by race/ethnicity for high-risk CHA_2_DS_2_-VASc group. *P* values were derived from logistic regression adjusted for age, sex, insurance type, and HAS-BLED score.

The association between race/ethnicity and rate of OAC prescription by low and high CHA_2_DS_2_-VASc risk groups is shown in **Table 3**. In the low-risk CHA_2_DS_2_-VASc group, only Asian (aOR, 0.64; 95% CI, 0.49–0.84) individuals with AF had a lower rate of OAC use compared to white individuals with AF **(Figure 2A)**. In the high-risk CHA_2_DS_2_-VASc group, only black (aOR, 0.89; 95% CI, 0.82–0.98) individuals with AF had a lower rate of OAC use compared to white individuals with AF **(Figure 2B)**.

**Table 3:** Association Between Race/Ethnicity and Use of Any Oral Anticoagulation by Low and High CHA₂DS₂-VASc Risk Groups

Race/Ethnicity	No. of Patients Using OACs (%)	Unadjusted Logistic Regression			Adjusted Logistic Regression		
		OR	95% CI	*P* Value	OR	95% CI	*P* Value
Low CHA₂DS₂-VASc Score (0–1 Points)							
White (n = 14,300)	4844 (33.9%)	1.00	(Reference)	—	1.00	(Reference)	—
Black (n = 451)	113 (25.1%)	0.65	0.53–0.81	<.001	0.93	0.74–1.17	.516
Hispanic (n = 653)	159 (24.4%)	0.62	0.52–0.74	<.001	0.97	0.80–1.18	.784
Asian (n = 338)	75 (22.2%)	0.56	0.44–0.73	<.001	0.64	0.49–0.84	.001
High CHA₂DS₂-VASc Score (2–9 Points)							
White (n = 86,962)	58,407 (67.2%)	1.00	(Reference)	—	1.00	(Reference)	—
Black (n = 2286)	1422 (62.2%)	0.81	0.74–0.88	<.001	0.89	0.82–0.98	.011
Hispanic (n = 2515)	1636 (65.1%)	0.93	0.86–1.01	.092	1.03	0.95–1.13	.447
Asian (n = 1494)	961 (64.3%)	0.89	0.81–0.99	.0375	0.92	0.82–1.02	.107

*Abbreviations:* CI, confidence interval; OAC, oral anticoagulant; OR, odds ratio. Logistic regression adjusted for age, sex, insurance type, and HAS-BLED.

**Figure 3:**
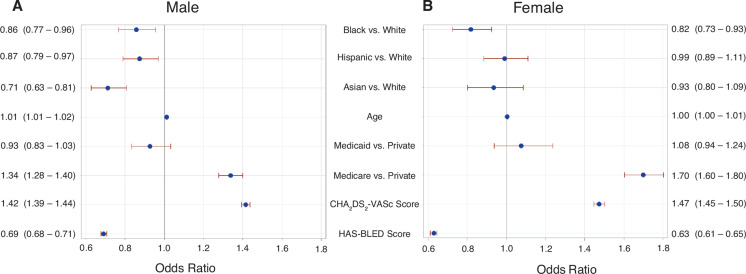
**A:** Odds ratio plot for male sex with 95% confidence intervals for adjusted logistic regression of being on anticoagulation by race/ethnicity. **B**: Odds ratio plot for female sex with 95% confidence intervals for adjusted logistic regression of being on anticoagulation by race/ethnicity. Both models adjusted for age, insurance type, CHA_2_DS_2_-VASc score, and HAS-BLED score.

The association between race/ethnicity and OAC use by sex is shown in **Table 4**. Within the male subgroup, black (aOR, 0.86; 95% CI, 0.77–0.96), Hispanic (aOR, 0.87; 95% CI, 0.79–0.97), and Asian (aOR, 0.71; 95% CI, 0.63–0.81) patients with AF were less likely to be on OACs compared to white patients with AF **(Figure 3A)**. Within the female subgroup, only black patients (aOR, 0.82; 95% CI, 0.73–0.93) with AF were less likely to be on OACs compared to white patients with AF **(Figure 3B)**.

**Table 4:** Association Between Race/Ethnicity and Use of any Oral Anticoagulation Stratified by Sex

Race/Ethnicity	No. of Patients Using OACs (%)	Unadjusted Logistic Regression			Adjusted Logistic Regression		
		OR	95% CI	*P* Value	OR	95% CI	*P* Value
Male Sex							
White (n = 59,598)	36,826 (61.8%)	1.00	(Reference)	—	1.00	(Reference)	—
Black (n = 1495)	822 (55.0%)	0.76	0.68–0.84	<.001	0.86	0.77–0.96	.006
Hispanic (n = 1695)	922 (54.4%)	0.75	0.68–0.82	<.001	0.87	0.79–0.97	.010
Asian (n = 1053)	568 (53.9%)	0.79	0.65–0.83	<.001	0.71	0.63–0.81	<.001
Female Sex							
White (n = 41,664)	26,425 (63.4%)	1.00	(Reference)	—	1.00	(Reference)	—
Black (n = 1242)	713 (57.4%)	0.78	0.70–0.87	<.001	0.82	0.73–0.93	.001
Hispanic (n = 1473)	873 (59.3%)	0.85	0.77–0.95	.003	0.99	0.89–1.11	.890
Asian (n = 779)	468 (60.1%)	0.88	0.76–1.02	.080	0.93	0.80–1.09	.377

*Abbreviations:* CI, confidence interval; OAC, oral anticoagulant; OR, odds ratio. Logistic regression adjusted for age, insurance type, CHA_2_DS_2_-VASc, and HAS-BLED.

Among patients on an OAC, only black (aOR, 0.83; 95% CI, 0.73–0.94) individuals with AF were less likely to be on a DOAC over warfarin compared to white individuals with AF **(Table 5)**.

**Table 5:** Association Between Race/Ethnicity and Use of Direct Oral Anticoagulants Over Warfarin

Race/Ethnicity	No. of Patients Using DOACs (%)	Unadjusted Logistic Regression			Adjusted Logistic Regression		
		OR	95% CI	*P* Value	OR	95% CI	*P* Value
White (n = 63,251)	50,339 (79.6%)	1.00	(Reference)	—	1.00	(Reference)	—
Black (n = 1535)	1203 (78.4%)	0.92	0.81–1.03	.174	0.83	0.73–0.94	.003
Hispanic (n = 1795)	1462 (81.4%)	1.12	0.99–1.26	.060	1.02	0.90–1.15	.740
Asian (n = 1036)	864 (83.4%)	1.29	1.09–1.51	.003	1.16	0.98–1.37	.087

*Abbreviations:* BMI, body mass index; CI, confidence interval; DOAC, direct oral anticoagulant; OR, odds ratio. Logistic regression adjusted for age, sex, insurance type, and BMI.

## Discussion

In this large study including 108,999 individuals with AF, we present several key findings that add to the mounting evidence that both racial/ethnic and sex disparities exist in the use of OACs. To the best of our knowledge, this is the first study to demonstrate that not only are women less likely to be prescribed OACs compared to men, but also black women are less likely to be prescribed OACs compared to their white counterparts. This finding suggests a compounding effect of female sex and black race that may result in a “double hit” that places black women at particularly high risk for undertreatment with OACs. Conversely, Hispanic and Asian women were found to have rates of OAC use comparable to that of white women, indicating that their race/ethnicity does not further contribute to undertreatment compared to white women. Meanwhile, Black, Hispanic, and Asian men had lower OAC prescription rates compared to white men, suggesting that race/ethnicity likely drives undertreatment in these groups. While this study is unable to demonstrate why black women are particularly undertreated with OACs, prescriber bias could be a contributing factor. Prior research on subjective clinical decision-making has shown that black women are uniquely subjected to undertreatment and were least likely to be referred for other cardiac therapies such as cardiac catheterization.^16^

As demonstrated in **Figure 1**, this study shows that black, Hispanic, and Asian individuals are less likely to be on OACs compared to white individuals. This study mirrors the growing body of evidence that all black patients are less likely to be prescribed OACs compared to white patients, though prior research on Hispanic and Asian patients has been mixed.^17–21^ These mixed results may be partially explained by the difficulty in categorizing Hispanic as a combined group (racial/ethnic) versus two unique entities (racial or ethnic). As mentioned in the Methods section, we chose to identify Hispanic as a combined group in accordance with the Executive Office of Budget and Management guidance to directly compare Hispanic with other racial groups.

This study finds that racial/ethnic disparities in OAC use are likely influenced by CHA_2_DS_2_-VASc risk groups. In low-risk groups, only Asian patients had lower OAC use, with age adjustment strongly impacting the group, as black and Hispanic patients were nearly 10 years younger than their white counterparts, as seen in **Supplementary Table S2**. In the high-risk group, only black individuals had lower OAC use compared to white individuals. This contrasts with the results reported by Essien et al., who found that black and Hispanic patients were less likely to be discharged on OACs following hospitalization for AF in both low and high CHA_2_DS_2_-VASc risk groups.^21^ Interestingly, in our study, 34% of white patients in the low-risk group used OACs, which is high considering that current 2019 AF guidelines have a IIA recommendation to consider omitting anticoagulation in this group.^7^ We propose several possible explanations for these findings, visually depicted in **Figure 2A**. First, it is possible that there is a prescriber bias toward the positive intervention of prescribing an OAC in white patients. While, strictly speaking, prescribing an OAC in a low-risk patient is not outside guidelines, it is not unreasonable to think that overtreatment may be occurring in this group given that studies have shown that white patients are more likely to receive an out-of-guideline cardiac intervention compared to other groups.^22^ Second, given the nuances around anticoagulating a low-risk patient, we posit that shared decision-making plays a strong role in treating this group. It is possible that shared decision-making is initiated less frequently or differs for black, Hispanic, and Asian patients, potentially due to varying levels of patient–physician trust, contributing to differential preferences in groups. This idea is supported by prior research showing that physicians engage in less shared decision-making with black patients.^23^

Variable rates of OAC use by race based on CHA_2_DS_2_-VASc risk groups have major implications for research in this domain. When analyzed in aggregate, it is possible that higher rates of OAC use in low-risk white patients contribute to the relative undertreatment of black, Hispanic, and Asian patients, as seen in **Figure 1**. However, when only high-risk patients are evaluated, only black individuals are shown to be undertreated compared to white individuals. This finding should be interpreted with caution as it is possible that Hispanic and Asian patients do in fact face racial/ethnic disparities in OAC use that are not captured in these data. However, it does add a level of nuance to the conversation around treatment equity as these data show that high-risk black patients are undertreated, while low-risk white patients may be exposed to higher bleeding risks via overtreatment.

Finally, this study reiterated established findings that black individuals were less likely to be on DOACs compared to white individuals.^8^ It did not show a difference in DOAC use between Hispanic and white patients, where again prior research has shown mixed results.^19,24^ This study did not show a difference in DOAC use between Asian and white patients, and there is limited prior research comparing these groups.

This study demonstrates clear differences in the use of OACs and DOACs by race/ethnicity and sex. Rathore and Krumholz provide a valuable framework for thinking about difference, disparity, and bias.^25,26^ A disparity is a difference of clinical consequence that exists after considering eligibility, contraindication, confounders, and patient preferences. While this study is unable to account for patient preference, it does meet the other criteria to demonstrate both racial/ethnic and sex disparities in the management of AF. Bias, defined as any decision-making principally based on race/ethnicity or sex, is difficult to study and beyond the scope of this investigation, though a large body of literature supports the notion that physicians hold bias, which influences clinical decision-making on the basis of race.^16,27^ The design of this study prevents us from specifically addressing these.

This study touches on racial/ethnic and sex disparities in OAC use and raises questions about OAC overuse in low-risk white patients. In the 2024 European Society of Cardiology guidelines on AF, it is recommended to reevaluate the inclusion of female sex as a standalone stroke risk factor in the CHA_2_DS_2_-VASc score. This change is based on emerging evidence that female sex does not consistently elevate stroke risk, especially in younger women without other cardiovascular risk factors. Should other professional societies adopt similar recommendations and remove sex as a stroke risk factor, it will be worth evaluating how this changes physician practice patterns and impacts the differential use of OAC in women, particularly black women.^28^

This analysis highlights an opportunity to improve the underuse of OACs in certain populations, with prior studies demonstrating that targeted implementation and intervention strategies can meaningfully reduce cardiac disease disparities.^29^ Some hospital systems have used population health managers and advanced electronic record systems such as digital dashboards to identify patients who may benefit from OACs; such systems could similarly be used to target at-risk racial/ethnic groups.^30^ Increasing shared decision-making through the use of a standardized tool is another promising strategy that could potentially improve OAC use by reducing bias in the shared decision-making process.^31^ Similarly, physician-based anticoagulation decision support tools also offer an opportunity to reduce bias in prescriber practice patterns, and there have been proposals to incorporate individual patients’ values into these support tools to better facilitate shared decision-making.^32^ In the inpatient setting, hospitals could institute a pharmacy-driven process whereby every new patient with AF receives appropriate risk counseling and treatment options for anticoagulation.^33^

### Limitations

First, all data came from a single hospital system, Mass General Brigham, and it will be important to confirm that similar results are seen across other institutions or national database registries. Despite an absolute number of black, Hispanic, and Asian patients comparable to other similar large studies, there was a relatively small percentage of these groups compared to white patients, which raises concerns about generalizability. Specifically, the fact that the racial and ethnic breakdown of the Mass General Brigham population used in this study differs from the demographics of the larger Boston area suggests a possible selection bias driven by racial and ethnic differences in access to care. We were unable to adjust for socioeconomic factors such as family income, level of education, or zip code demographics. While we were able to adjust for some health system factors, such as insurance type, the data did not provide the granularity needed to address confounders like differences in disposable income or variations in co-pays and deductibles within a plan that influence a patient’s medication cost burden.

This study relied on electronic medical record (EMR) data, which have inherent limitations, and prior studies have specifically demonstrated that misdiagnosis of AF is common in EMR due to false-positive or inactive AF diagnoses.^12^ While all patients in this study had a presumed diagnosis of AF, we were not able to identify patients who had additional diagnoses and potentially other indications for OACs. Similarly, when comparing DOAC therapy versus warfarin, we were not able to identify patients who may have had an indication specifically for warfarin. We were also not able to identify patients who may have had a reduced stroke risk based on prior interventions such as left atrial appendage closure or occlusion devices. Lastly, this study relied on active orders in the EMR medication list to determine anticoagulation status, which does not capture patient medication adherence or proper medication use. We were also unable to assess the degree to which racial/ethnic differences in OAC use were driven by differing preferences around the risks/benefits of anticoagulation.

## Conclusions

This study demonstrates the novel finding that not only are women less likely to be on OACs compared to men, but also black women face an additional disparity when compared to their white counterparts. This study also found that there are variable rates of OAC use by race, based on CHA_2_DS_2_-VASc risk groups. Specifically, in the high-risk group, only black individuals are less likely to be on OACs compared to white individuals. Additionally, low-risk white patients have relatively high rates of OAC use, raising questions about prescriber bias or differential shared decision-making in this group. Lastly, this study confirms prior studies showing that black, Hispanic, and Asian patients are less likely to be on OACs and that black patients are less likely to be on DOACs.
